# Antibacterial and Antibiofilm Efficacy of Phenyllactic Acid Against Foodborne Pathogens *Salmonella enterica* Serotype Derby and *Escherichia coli* O26

**DOI:** 10.3390/molecules30081738

**Published:** 2025-04-13

**Authors:** Angela Maione, Annalisa Buonanno, Marianna Imparato, Giuseppe Maglione, Cristina Rossetti, Angela Michela Immacolata Montone, Marco Guida, Emilia Galdiero, Paola Zinno

**Affiliations:** 1 Department of Biology, University of Naples “Federico II”, 80126 Naples, Italy; angela.maione@unina.it (A.M.); annalisa.buonanno@unina.it (A.B.); marianna.imparato@unina.it (M.I.); marco.guida@unina.it (M.G.); 2 Institute for the Animal Production System in the Mediterranean Environment (ISPAAM), National Research Council, Piazzale Enrico Fermi 1, 80055 Portici, Italy; giuseppe.maglione@cnr.it (G.M.); cristina.rossetti@cnr.it (C.R.); paola.zinno@cnr.it (P.Z.); 3 Department of Food Inspection, Istituto Zooprofilattico Sperimentale del Mezzogiorno, Via Salute 2, 80055 Portici, Italy; angela.montone@izsmportici.it; 4 BAT Center-Interuniversity Center of Studies on Bioinspired Agro-Environmental Technology, University of Naples Federico II, 80055 Portici, Italy

**Keywords:** phenyllactic acid, antibacterial agent, biofilm, natural products, foodborne illness pathogens, food safety

## Abstract

Nowadays, the spread of foodborne diseases and the growing concerns about antibiotic resistance have shifted the focus of researchers towards the use of substances of natural origin. Phenyllactic acid (PLA), a naturally produced compound, has already demonstrated antimicrobial properties against pathogenic microorganisms and those responsible for food spoilage. This study examines the antibacterial and antibiofilm properties of PLA against foodborne pathogens such as *Salmonella enterica* Derby and *Escherichia coli* O26. The study showed that PLA effectively inhibited both biofilm formation and bacterial planktonic growth, with minimal inhibitory concentrations (MICs) ranging from 2 to 2.75 mg mL^−1^. A dose-dependent inhibition of biofilm formation was observed, reaching approximately 90% for *Salmonella* strains and 50% for *E. coli* at 1.5 mg mL^−1^. The cytotoxicity evaluation on Caco-2 cells showed that PLA was well-tolerated at concentrations up to 2 mg mL^−1^. PLA’s effectiveness was also demonstrated in real food matrices, where its application in minced beef stored at 4 °C significantly reduced microbial populations, unlike in untreated samples where bacterial counts increased. PLA showed a good ability to inhibit biofilm formation and eradicate a mature biofilm, measuring the total bacterial biofilm biomass. Additionally, PLA was found to be biocompatible in Caco-2 cells, confirming that it poses no health risk at the tested concentrations. The study also observed that PLA reduced bacterial adhesion to intestinal cells, suggesting its potential in preventing intestinal bacterial colonization. These results highlight PLA as a promising natural antimicrobial agent for food preservation, with potential applications in sustainable packaging and controlling microbial contamination in food processing. Future studies should further explore PLA’s long-term stability and its interactions in complex food environments.

## 1. Introduction

The prevalence of foodborne diseases is a significant global health concern, with an estimated 600 million individuals (approximately 10% of the global population) falling ill from consuming contaminated food annually [1].

*Salmonella enterica* and shigatoxigenic *Escherichia coli* (STEC) are still emerging pathogens responsible for most cases of foodborne illness. The actual number of cases of foodborne illness is likely to be higher due to unrecognized or unreported outbreaks, leading to an increase in statistics. In 2021, Shiga toxin-producing *E. coli* (STEC) cases rose to over 6500, with Germany, Denmark, and Ireland reporting the most cases. Common *E. coli* serogroups included O157, O26, O103, and O145, which caused outbreaks linked to various food items such as raw milk and pre-cut vegetables [2,3].

In February 2024, a major outbreak of *E. coli* O26 was identified in France. The outbreak was traced to the consumption of raw milk cheese [4].

In 2022, salmonellosis was the second most frequently reported gastrointestinal infection from food in the European Union and significantly contributed to foodborne outbreaks across both EU member states and non-member state nations. The latest annual EU One Health Zoonoses Report published by the European Food Safety Authority (EFSA) and the European Centre for Disease Prevention and Control (ECDC) shows 65,208 cases, which is the same rate of notification as in 2021. The distribution of the top five *Salmonella* serovars acquired within the European Union and implicated in human infections was as follows: *Salmonella* Enteritidis, *Salmonella* Typhimurium, *Salmonella* Typhimurium monophasic variant, *Salmonella* Infantis, and *Salmonella* Derby. These serovars, as described for *S*. Infantis, may exhibit diverse genetic characteristics, including the presence of antimicrobial resistance genes, tolerance to heavy metals, mobile virulence genes, and the capacity to form biofilms. These characteristics could bolster their epidemiological resilience and confer additional virulence and persistence attributes within the food supply chain [5]. Monitoring *Salmonella* strains is of paramount importance, particularly in instances where these bacteria have acquired adaptations that enable them to thrive in challenging environmental settings [6]. A wide range of foodstuffs have been identified as potential vectors for *Salmonella* outbreaks. Poultry remains a significant contributor to the dissemination of this pathogen, but other animal-derived foods, such as beef and seafood, also play a role [7]. While beef products are less commonly linked to human infections compared to pork, they have been implicated in significant outbreaks caused by multidrug-resistant *Salmonella* in both Europe and North America [8,9].

Although pre-harvest interventions implemented at the farm level and during livestock transport aim to minimize the introduction, persistence, and transmission of *Salmonella* on farms [10], meat safety is significantly influenced by the bacterial load of animals upon arrival at the slaughterhouse and by procedures to mitigate cross-contamination during slaughter and meat processing [11]. Furthermore, *Salmonella* can adhere, proliferate, and establish biofilms in food matrices or on the surfaces of meat or meat products and their processing equipment, thereby resulting in persistent contamination [12]. The ability of foodborne strains to form biofilms on various surfaces increases their persistence in different environments by enhancing their resistance to physical and chemical stresses. Biofilm-forming bacteria can adhere to a variety of surfaces, including stainless steel, plastic, glass, and even food. This adhesion process takes only a few minutes, and mature biofilms can develop within hours or days, posing significant challenges for the food industry [13].

It has been observed that certain antimicrobial agents employed in food production to extend shelf life and enhance hygiene standards are now less efficacious than before due to the emergence of microorganisms resistant to these substances [14]. Consequently, there is a pressing need to discover novel antimicrobials capable of combating microorganisms that have developed resistance to conventional treatments. Furthermore, consumers today seek food free from artificial substances, such as antimicrobial agents and chemically synthesized preservatives, as these are viewed as potentially harmful to health. The growing demand for products without synthetic preservatives has driven the search for natural antimicrobial compounds. Extensive research has been conducted on natural food additives with a wide range of antimicrobial properties, capable of enhancing the microbiological quality and extending the shelf life of perishable foods [15].

Phenyllactic acid (PLA), a natural organic acid derived directly from phenylpyruvic acid, can be synthesized by specific strains of lactic acid bacteria using lactate dehydrogenase [16,17].

PLA is known for its effective antimicrobial properties, rendering it a crucial resource for food preservation, particularly in the context of biopreservation. This natural compound is produced by certain lactic acid bacteria (LAB) during the fermentation process. It has been demonstrated to be effective in combating a range of harmful microorganisms, including bacteria and fungi [18,19].

PLA has been demonstrated to be effective in prolonging the shelf life of food products by preventing microbial spoilage. Although the precise mechanism of action of PLA is not yet fully elucidated, studies have suggested that it may compromise the cell wall integrity of exposed bacteria [20,21]. This makes PLA particularly efficacious in the preservation of products such as dairy, meat, and bakery products, where microbial contamination is a common occurrence. Furthermore, the compound is being investigated for its potential use in active food packaging systems, which can create antimicrobial barriers on packaging surfaces. The use of PLA by the food industry is intended to mitigate the risk of foodborne diseases and extend the shelf life of products. This aligns with consumer demand for preservation solutions that utilize naturally derived ingredients as an alternative to synthetic additives.

The prevalence of foodborne pathogens such as *S. enterica* and *E. coli* in combination with their increasing resistance to conventional antimicrobial agents underscores the need for novel and sustainable solutions. PLA, a natural compound with potent antimicrobial and antibiofilm properties, is a promising candidate for enhancing food safety and preservation.

In this context, the present study aims to evaluate the in vitro antibacterial effects of PLA against important and common pathogens of foodborne infections. Although PLA is well-known for its ability to inhibit the growth of both Gram-negative and Gram-positive bacteria, there may be significant variability in its antibacterial efficacy due to the differential susceptibility of microbial isolates. This study expands on previous research by not only assessing the efficacy of PLA against these pathogens in both planktonic and biofilm forms, but also exploring its impact at the host–pathogen interface. Specifically, we investigated the ability of PLA to inhibit biofilm formation, reduce microbial viability, and ensure safety through cytotoxicity assessments. Additionally, we examined its effects on bacterial adhesion to intestinal epithelial cells and its potential role in preserving intestinal barrier integrity by modulating membrane junction permeability in Caco-2 cells. Exploring its applications in food matrices and its interactions with host cells will contribute to establishing PLA as an effective and sustainable alternative to traditional antimicrobial strategies, with promising implications for food preservation, microbial control, and food safety along the entire food chain.

## 2. Results

### 2.1. Phenotypic Antimicrobial Resistance Characterization

Antimicrobial susceptibility testing revealed notable differences in resistance profiles between *Salmonella enterica* serovar Typhimurium DSMZ 18,522 (S4), *Salmonella enterica* Derby (S1), and *Escherichia coli* O26 (Table 1). In particular, S1 and S4 showed resistance to ampicillin (AMP), with MIC values of 64 µg mL^−1^ and greater than 512 µg mL^−1^, respectively, both exceeding the European Committee on Antimicrobial Susceptibility Testing (EUCAST) resistance threshold (R > 8 µg mL^−1^). This widespread resistance to ampicillin highlights the potential challenge of treating infections caused by these serovars. Conversely, both S1 and S4 exhibited continued sensitivity to kanamycin (KANA), chloramphenicol (CHL), and tetracycline (TET). The minimum inhibitory concentrations (MICs) for these antibiotics were consistently low, with kanamycin and tetracycline documented at 4 µg mL^−1^, and chloramphenicol MICs also at 4 µg mL^−1^. These findings suggest that these antibiotics may still be viable treatment options against these Salmonella serovars. The E2 strain, however, exhibited a distinct multidrug-resistant profile, with resistance to all the four tested antibiotics, and an ampicillin minimum inhibitory concentration (MIC) of 16 µg mL^−1^, which exceeded the resistance threshold. Moreover, kanamycin and chloramphenicol MICs were exceptionally high, each greater than or equal to 512 µg mL^−1^, clearly indicating resistance. The MIC for tetracycline was elevated at 16 µg mL^−1^, which is greater than the EUCAST cut-off value. This pronounced multidrug resistance observed in E2 underscores its clinical significance and raises concerns about its role in difficult-to-treat infections.

### 2.2. Antibacterial Activity of PLA

The in vitro antibacterial activity of PLA was determined using quantitative assays. Table 2 shows the minimum inhibitory concentrations (MICs) and the minimum bactericidal concentrations (MBCs). The MIC was determined by using the microdilution method, and the results reported in Table 2 show MIC values of 2 and 2.5 µg mL^−1^ observed for the two *Salmonella* strains and 2.75 µg mL^−1^ for E2. The MBC of PLA was 5 mg mL^−1^ for S1 and S4 and 5.5 mg mL^−1^ for E2, demonstrating a bactericidal effect against all the three strains, with an MBC/MIC ratio < 4.

Considering the importance of *Salmonella* in foodborne infections, PLA could be considered a good green alternative.

### 2.3. Challenge Test

**Figure 1:**
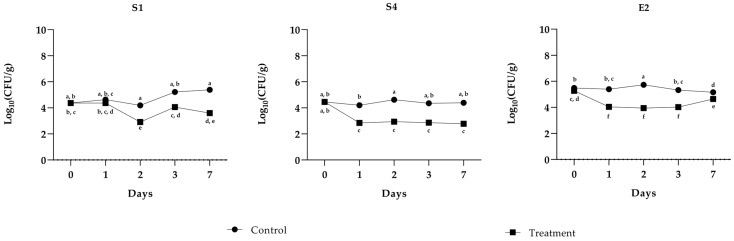
Challenge test of PLA against *S. enterica* Derby (S1), *S. enterica* Typhimurium DSMZ 18,522 (S4), and *E*. *coli* O26 (E2). The assay was performed in minced cow meat artificially contaminated with pathogens with or without the addition of PLA. The meat was stored at 4 °C for up to 7 days. Bacterial cell viability is expressed as the geometric mean of CFU g^−1^ SD of one experiment carried out in triplicate. Means without a common letter significantly differ, *p* < 0.05 (Tukey’s test).

The study evaluated the antimicrobial effect of PLA (1 µg mL^−1^) on S1, S4, and E2 in minced beef stored under refrigeration at 4 °C for up to seven days. The results, summarized in the graphs, highlight consistent trends for the pathogens and the impact of the PLA treatment over time (Figure 1). In untreated samples (inoculated with the indicator pathogen but not treated with PLA), the bacterial populations of all the three pathogens remained relatively stable throughout the storage period, with minimal fluctuations. For E2, the population consistently varied between 5 and 6 log CFU g^−1^, while S1 showed a similar trend, stabilizing around 5 log CFU g^−1^ with minimal fluctuations. In contrast, the samples treated with PLA exhibited a significant decrease in bacterial populations for all the tested pathogens. For E2, a notable decline was observed as early as day 1 (T1), with levels dropping to approximately 4 log CFU g^−1^ and maintaining stability until day 7. A comparable trend was observed in the case of S1, where PLA treatment led to a significant reduction in counts by T1, with levels dropping below 4 log CFU g^−1^ by T2 and remaining at low levels thereafter. The antimicrobial activity of PLA was consistent across pathogens. In the untreated samples, refrigeration alone was insufficient to suppress bacterial growth, as evidenced by stable or slightly increasing populations during storage. However, PLA treatment clearly demonstrated its efficacy in reducing bacterial counts and maintaining lower levels throughout the seven-day period. The control samples, which were neither inoculated nor treated with PLA, exhibited no bacterial growth, thereby confirming the absence of contamination by *Salmonella* and *Escherichia* bacteria in the minced meat utilized for the experiment. This further validates the efficacy of the prior thermal treatment in achieving complete inactivation of *E. coli*, ensuring the microbiological integrity of the samples. According to the USDA Food Safety and Inspection Service, ground meat should be cooked to a minimum internal temperature of 71 °C to ensure the elimination *of E. coli* [22].

### 2.4. Antibiofilm Activity of PLA

The ability of the collection strain and the two foodborne isolates to assemble biofilms in vitro after 24 h of incubation at 37 °C was evaluated. All the strains were considered biofilm producers according to Stepanovic’s classification [23]. S1 was considered a moderate biofilm producer, while S4 and E2 were classified as strong biofilm producers, as shown in Table 3.

**Figure 2:**
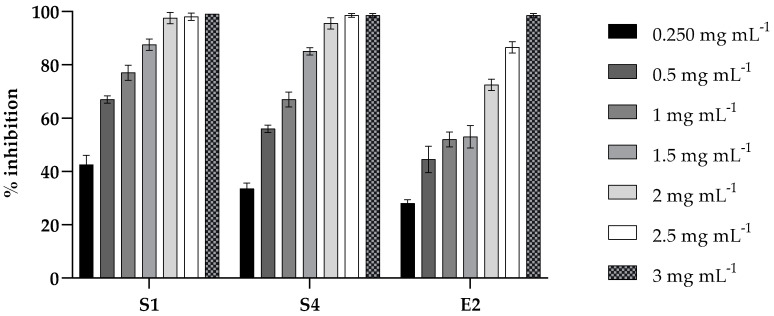
Inhibition effect of PLA on microbial biofilm formation in 96-well microplates. *S. enterica* Derby (S1), *S. enterica* Typhimurium DSMZ 18,522 (S4), and *E*. *coli* O26 (E2) were co-incubated with various concentrations (0.2, 0.5, 1, 1.5, 2, 2.5, and 3 mg mL⁻^1^) of PLA for 24 h and their biofilm production was compared to that of the cells incubated without PLA (CV assay). Values obtained are given as the percentage of biofilm formation. The results shown represent the means and standard deviations (error bars) of three independent experiments.

The inhibition of biofilm formation by PLA was evaluated at different concentrations ranging from 0.25 to 3 mg mL^−1^ against strains S1, S4, and E2. As shown in Figure 2, a dose-dependent inhibitory effect was observed for all the strains. At the sub-MIC of 1.5 mg mL^−1^, an inhibition of biofilm formation of about 90% for *Salmonella* strains (S1 and S4) and of about 50% for E2 was observed.

**Figure 3:**
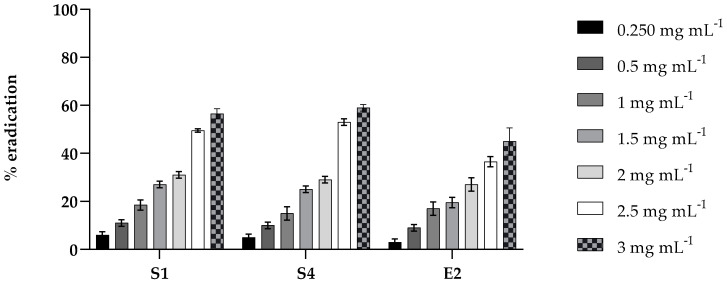
Eradication effect of PLA on mature biofilms in 96-well microtiter plates. Mature biofilms of *S. enterica* Derby (S1), *S. enterica* Typhimurium DSMZ 18,522 (S4), and *E*. *coli* O26 (E2) were treated with various concentrations (0.2, 0.5, 1.0, 1.5, 2, 2.5, and 3 mg mL^⁻1^) of PLA for 24 h. Values obtained are given as the percentage of biofilm eradication. The results shown represent the means and standard deviations (error bars) of three independent experiments.

The effect of PLA was also tested on the mature biofilm (48 h) of S1, S4, and E2 at the same concentration. The results, shown in Figure 3, exhibit moderate activity in eradicating the biofilm of the three strains, reaching approximately 50% at the highest concentration tested.

### 2.5. Cytotoxicity Assays

**Figure 4:**
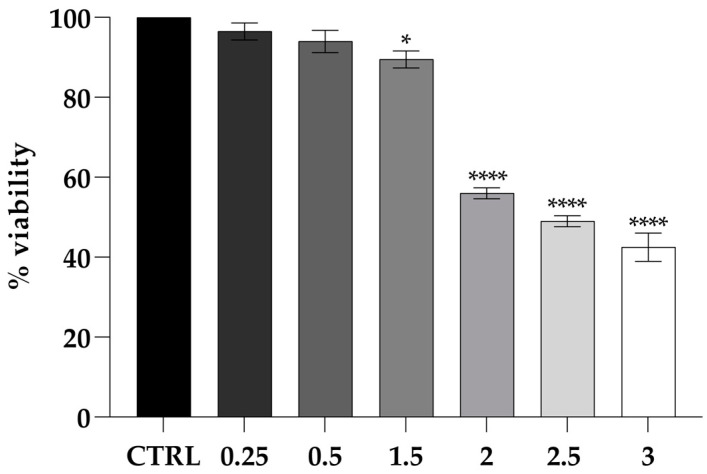
Viability of the CaCo-2 cell line challenged by PLA shown as the percent cell viability of the cells treated with different concentrations for 24 h. Untreated cells were used as the control. The assays were performed in three independent experiments. One-way ANOVA, followed by Dunnett’s test, was performed to determine statistically significant results. Note: * = *p* < 0.05, **** = *p* < 0.0001.

The cytotoxicity of PLA was evaluated at concentrations of 0.25, 0.5, 1, and 2 mg mL^−1^ (Figure 4). Cell viability persisted, indicating no cytotoxicity at low concentrations. However, at 3 mg mL^−1^, a significant reduction in cell viability was observed (****, *p* < 0.0001), suggesting a cytotoxic effect at higher concentrations.

### 2.6. Adhesion of Enteric Bacteria to Caco-2 Cells with PLA Treatments

**Figure 5:**
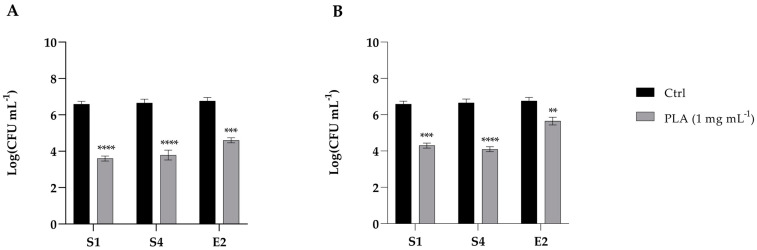
Inhibition of the adhesion of *S. enterica* Derby (S1), *S. enterica* Typhimurium DSMZ 18,522 (S4), and *E*. *coli* O26 (E2) to CaCo2 cells pre-treated (**A**) or post-treated (**B**) with PLA at a concentration of 1 mg mL^−1^. Data reported are the means of three independent experiments ± SD. The statistical significance with respect to the corresponding controls is indicated by ** *p* < 0.001, *** *p* < 0.0001, and **** *p* < 0.00001 (Tukey’s test).

The ability of PLA to inhibit the adhesion of S1, S4, and E2 bacteria to intestinal cells was evaluated by pre- and post-treating CaCo-2 with 1 mg mL^−1^ of PLA. As shown in Figure 5 (panel A), the pre-treatment with PLA significantly reduced the adhesion of S1, S4, and E2 by about 2 log. An adhesion reduction of 2 log was also observed for the post-treatment of S1 and S4 with PLA, and of only about 1 log for E2 (Figure 5B). Altogether, these data suggest that PLA is nontoxic and shows potential for preventing enteric bacterial interaction with intestinal cells at the tested doses.

### 2.7. Effects of PLA on Caco-2 Cell Permeability

**Figure 6:**
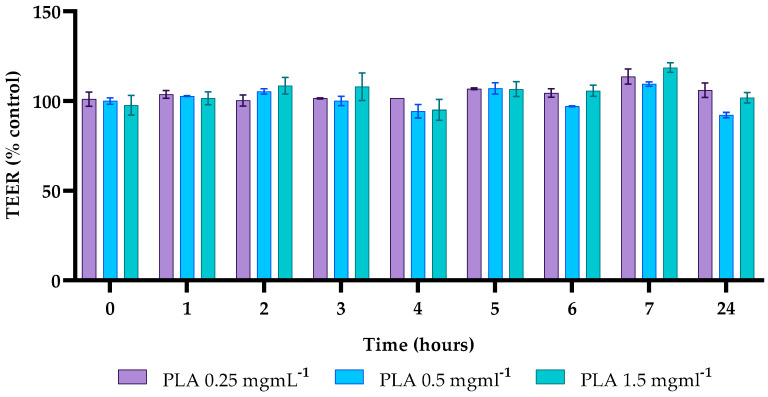
Transepithelial electrical resistance (TEER). TEER values for different concentrations of PLA, recorded for up to 24 h, were calculated as the percentage of C for each timepoint.

In this study, the integrity of the intestinal barrier of differentiated Caco-2 cells, treated with different concentrations of PLA (0.25, 0.5, 1.5 mg mL^−1^) was studied by TEER measurement. TEER values at 1, 2, 3, 5, 7, and 24 h post-treatment remained higher than the control value, except for the 0.5 mg mL^−1^ concentration, where TEER values were 92% of the control (Figure 6).

The obtained data indicate that PLA does not induce any damage to the integrity of the intestinal barrier of Caco-2 cells at the concentrations and times tested.

## 3. Discussion

This study investigated the antibacterial and antibiofilm activity of PLA, a naturally occurring organic acid, against foodborne pathogens, including a collection strain *Salmonella enterica* serovar Typhimurium and two isolates belonging to *Salmonella enterica* Derby and *Escherichia coli* O26 [24]. Salmonellosis remains the second most common foodborne zoonotic disease in the EU, with *S. typhimurium* strains primarily originating from various sources, including broilers, pigs, laying hens, turkeys, and cattle [25]. Similarly, *E. coli* serogroup O26 has been identified as the second most prevalent STEC-related foodborne disease in a number of European countries, including Ireland, Italy, France, and Denmark [26]. The results obtained in this work are particularly relevant considering the increasing prevalence of foodborne diseases and the rise of antimicrobial resistance among these pathogens. Tests conducted on these isolates exhibited resistance profiles and strong biofilm-forming capabilities. For instance, the *Salmonella* Derby isolate demonstrated a strong ability to form a biofilm, which can facilitate its persistence on food processing surfaces and within the food matrix. Similarly, the *E. coli* O26 isolate presented a multidrug-resistant phenotype, raising significant public health concerns. Application of PLA resulted in a marked reduction in both planktonic and biofilm-associated bacterial growth for these isolates. A salient finding of this study is the inhibitory effect of PLA on planktonic cells and biofilm formation. In vitro assays demonstrated that the minimum inhibitory concentrations (MICs) for the tested strains ranged from 2 to 2.75 mg mL^−1^, exhibiting comparable minimum bactericidal concentrations (MBCs). These findings suggest that PLA has a broad spectrum of activity, which is consistent with previous studies that have highlighted its antimicrobial properties against both Gram-positive and Gram-negative bacteria [16,17]. Furthermore, PLA’s ability to reduce biofilm formation and act on mature biofilms indicates its potential use for controlling persistent microbial contamination, a relevant aspect in food processing and preservation. Recent studies have shown PLA’s strong anti-biofilm properties against microorganisms such as *Enterobacter cloacae*, *Pseudomonas aeruginosa*, *Listeria monocytogenes*, and *Enterococcus faecalis*. In particular, PLA has been shown to reduce biofilm formation even at subinhibitory concentrations and disrupt biofilms by damaging cell membranes and causing intracellular leakage. PLA also demonstrates synergy with slightly acid-electrolyzed water to enhance its effects against bacteria such as *Klebsiella oxytoca*. Additionally, PLA has been shown to interfere with the quorum-sensing system of bacteria and inhibit the expression of virulence factors, such as pyocyanin and protease, which are involved in biofilm formation [27]. The challenge test performed on minced beef stored at 4 °C provided practical indications on the applicability of PLA in realistic food matrices. In contrast to the untreated samples, in which the bacterial count remained stable or even slightly increased during cold storage, the PLA-treated samples showed a significant reduction in microbial populations over a seven-day period. As a case in point, the load of E2 decreased to approximately 4 log CFU g^−1^ at T1, while S1 decreased below 4 log CFU g^−1^ at T1 and remained consistently low throughout the 7-day storage period. A similar result was reported in [28], where PLA was used to reduce the presence of Gram-positive bacteria. The authors reported that the addition of PLA at a concentration of 3 mgL^−1^ to UHT and pasteurized whole milk effectively inhibited the growth of *L. monocytogenes* for a 14-day storage period at 4 °C. Furthermore, the study showed a reduction in the *L. monocytogenes* load on the surface of spiced beef treated with PLA at concentrations ≥3 mgL^−1^ during the storage period. By evaluating PLA when used to treat Gram-negative bacteria, our study expands its potential antimicrobial spectrum. Despite employing an approach divergent to that adopted in our study, further evidence of the effectiveness of PLA in ensuring food safety is provided in [29], which describes the development of an innovative food packaging based on an antibacterial hydrogel of gelatin, chitosan, and 3-phenyllactic acid, called GCP-1, designed to extend the shelf life of chilled chicken. The GCP-1 antibacterial formulation exhibited effective antimicrobial activity against both *Staphylococcus aureus* and *Escherichia coli*, thereby significantly extending the shelf life of the product. Since high concentrations of PLA have been shown to result in the production of a pungent odor, the concentration that was tested in the present study may be considered optimal in order to achieve the desired antimicrobial effect while maintaining the organoleptic properties of the food. This decrease in the pathogenic load not only validates the antimicrobial effectiveness of PLA, but also indicates that its incorporation into storage methodologies could potentially extend the shelf life of perishable products, contributing to enhanced food safety. The composition of ground beef necessitates consideration of its substantial fat content; this can result in the potential for enhanced bacterial resistance, thereby mitigating the effect of stress on bacterial populations. Consequently, the employment of a combination of diverse antibacterial agents within food applications can serve to counterbalance this effect, thereby achieving a more pronounced decontamination outcome. The antimicrobial mechanism of PLA, although not fully elucidated in this study, is likely to be related to its ability to disrupt bacterial cell membranes and interfere with key cellular processes. It is well-established that PLA exhibits antimicrobial properties; however, the precise mechanism through which it functions remains to be elucidated. Ning et al. [30] proposed that the amphiphilic nature of PLA enables its interaction with membrane lipids and proteins, thereby affecting their permeability and integrity. This mechanism is analogous to that observed in other phenolic acids [20]. However, future investigations should aim to further elucidate these mechanisms, particularly in the context of complex food matrices where interactions with other components may occur.

PLA represents a promising agent for the control of pathogenic biofilms in food processing environments, although its efficacy may vary depending on the target microorganism. The study by Liu et al. [31] showed high activity of PLA as a sanitizer against *Listeria monocytogenes*, an efficacy that appears to be higher than that found in our study against *Salmonella* and *E. coli*. This difference could be attributed to the distinct composition of the biofilm extracellular matrix among the bacterial species, which may influence the efficacy of PLA in penetrating and disrupting the biofilm structure.

Safety assessments were conducted on Caco-2 cells, a highly used model in part due to their ability to reproduce in vitro the intestinal mucosa environment and as such provide useful data on both the possible inflammatory state or alteration generated by pathogens [32]. Indeed, the intestinal lining acts as a protective barrier, due to the presence of tight junctions (TJs) located in the apical region of the epithelial cells’ lateral membrane. This barrier is capable of protecting the organism from pathogens or allergens, yet allowing the passage of water and ions. Damage to the integrity of this barrier determines an alteration of the normal functions of the intestinal barrier [24]. In this study, cytotoxicity assays showed that PLA, even at concentrations up to 2 mg mL^−1^, does not adversely affect cell viability. Furthermore, transepithelial electrical resistance (TEER) measurements confirmed that exposure to PLA at three different concentrations (0.25, 0.5, and 1.5 mg mL^−1^) did not determine any alteration of the integrity of the intestinal barrier, a crucial factor in ensuring the safe interaction of food packaging materials with the human digestive system. These results are significant, as they indicate that PLA can be safely incorporated into food systems and active packaging without posing a health risk to consumers. Given the growing demand for sustainable and biodegradable packaging solutions, the proven biocompatibility of PLA reinforces its validity as a sustainable alternative to conventional strategies adopted in the food industry.

The inhibitory effect of PLA is also shown on the adhesion of enteric bacteria (S1, S4, and E1) to intestinal epithelial cells, both before and after treatment with 1 mg mL^−1^ PLA. These results suggest that PLA may serve as an effective antimicrobial agent to reduce bacterial colonization in the intestinal environment. This suggests that PLA could potentially create a protective barrier or induce changes in the cell surface, reducing bacterial attachment. Potential mechanisms could include modulation of host cell receptors or the formation of a protective layer by interacting with the cell microenvironment, thereby changing the physicochemical properties of the epithelial surface and rendering it less hospitable for bacterial adhesion [17].

Jiang et al. reported that, despite its strong antibiofilm properties, PLA did not significantly affect *L. monocytogenes* adhesion and invasion in Caco-2 cells. In contrast, our study demonstrated that PLA effectively reduced the adhesion of *Salmonella enterica* and *E. coli* O26 to Caco-2 cells, suggesting that its antimicrobial interactions may vary depending on the bacterial species. The observed discrepancy could be ascribed to variations in bacterial surface structures, adhesion strategies, or PLA’s capacity to interfere with specific virulence factors in Gram-negative versus Gram-positive bacteria.

## 4. Materials and Methods

### 4.1. Strain and Culture Conditions

The microorganisms used were *Salmonella enterica* serovar Typhimurium DSMZ 18,522 (DSMZ; Braunschweig, Germany) (S4), while the isolates of *Salmonella enterica* Derby (S1) from chicken samples and *Escherichia coli* O26 (E2) provided by the Istituto Zooprofilattico Sperimentale del Mezzogiorno (Portici, Naples, Italy). *Salmonella* strains were routinely grown in tryptone soy broth (TSB; Oxoid, Basingstoke, UK) while *E. coli* O26 (E2) was grown in Luria–Bertani (LB) broth, Miller (DIFCO, Rodano (MI), Italy) at their optimal growth temperature, 37 °C.

### 4.2. Antibiotic Susceptibility Test

Antibiotic susceptibility test was performed for a selected panel of antibiotics, namely ampicillin, kanamycin, erythromycin, chloramphenicol, and tetracycline chosen as representatives of the most used pharmacological classes of antimicrobials.

The minimum inhibitory concentration (MIC) evaluation was performed using the broth microdilution method in accordance with internationally recognized standard protocols from the Clinical and Laboratory Standards Institute (CLSI) and ISO standards. The antibiotic concentrations tested ranged from 1 to 512 mg L^−1^ for all the antibiotics and from 2 to 512 mg L^−1^ for tetracycline. The antibiotics were provided by Sigma-Aldrich (Milan, Italy). Resistance profiles were generated, with isolates classified as multidrug-resistant (MDR) if they showed non-susceptibility to one antimicrobial agent in three or more antimicrobial classes [33,34].

### 4.3. Determination of the Minimum Inhibitory Concentration (MIC) and of the Minimum Bactericidal Concentration (MBC) of PLA

The MIC of PLA was determined according to the CLSI guidelines [34]. PLA (Sigma-Aldrich, Saint Louis, MO, USA) was dissolved in DMSO 5% *v*/*v* to prepare a 50 mg mL^−1^ stock solution. Briefly, the bacterial culture was diluted to a final concentration of 1 × 10^6^ cells mL^−1^ in TSB (Oxoid, Basingstoke, UK) at 37 °C for 24 h together with PLA ranging from 0.625 to 10 mg mL^−1^, and growth was determined at the 590 nm wavelength with a microplate reader (Varioskan LUX Multimode Microplate Reader; Thermofisher Scientific, Waltham, MA, USA). The MIC was defined as the lowest drug concentration that resulted in a ≥90% inhibition of growth compared to the growth control.

Following the MIC assay, the minimum bactericidal concentration (MBC) of PLA was determined. Ten μL of the sample from each well without visible growth were plated on tryptone soy agar plates (TSA; Oxoid, Basingstoke, UK) and incubated at 37 °C for 24 h. The MBC was defined as the lowest concentration of PLA that completely inhibited bacterial growth after plating. The compound was considered bactericidal if the MBC/MIC ratio was ≤4, and bacteriostatic if the ratio was ≥4.

### 4.4. Challenge Test

The challenge test used minced beef from a supermarket. Meat portions (100 g each), previously heat-treated at 72 °C for 20 s, were inoculated with an S1, S4, or E2 inoculum (approximately 1 × 10^4^ CFU g^−1^), as described in Serio 2010 [35].

To ensure effective pathogen distribution, the inoculated samples were homogenized using Stomacher bags (Bag Mixer-400; Interscience, Saint-Nom-la-Bretèche, France) for 2 min at room temperature. After homogenization, the inoculated meat was divided into two equal portions (50 g each), one of which was treated with PLA at a concentration of 1 mg mL^−1^. In addition, one meat portion was neither inoculated nor PLA-treated (control). The Stomacher bags were sealed and stored under aerobic conditions at 4 °C for up to seven days.

Microbiological analysis of the populations of the S1, S4, and E2 isolates was carried out after 0–1–2–3 and 7 days of refrigerated storage. At each sampling time, the aliquots of minced meat were aseptically diluted 10-fold in 0.9% NaCl and homogenized for 2 min at room temperature in Stomacher bags. The resulting slurries were serially diluted and plated in duplicate on a selective xylose lysine deoxycholate (XLD) agar (Merck, Darmstadt, Germany). The populations of bacteria were determined and quantified by means of colony counting, after incubation for 24 h.

Before inoculation with the pathogens, the minced meat was also examined for the presence of any bacteria by means of estimating the total viable counts (TVCs) on plate count agar (PCA; Oxoid, Basingstoke, UK) after 72 h of incubation at 25 °C.

### 4.5. Biofilm Formation

The biofilm-forming potential of the strains used in this study was investigated using our previous protocol [36]. Briefly, to form biofilms, a 96-well sterile flat-bottomed microplate was seeded with a 10^6^ CFU mL^−1^ inoculum of the test organisms (100 μL per well) and incubated for 24 h at 37 °C. The total biofilm mass was detected using the crystal violet (CV; Sigma-Aldrich, Saint Louis, MO, USA) staining methodology. After incubation, the plate was washed three times with phosphate-buffered saline (PBS; Oxoid, Basingstoke, UK), and the biofilm was fixed at 37 °C for 1 h. The CV solution (0.2% *v*/*v*) was added to each well (200 µL), and after 15 min of incubation at room temperature, the excess was washed with PBS. The bound CV was solubilized with 300 µL of 30% (*v*/*v*) acetic acid, and absorbance was measured at 570 nm using a microplate reader [37]. OD cut-off (ODc) was calculated to classify the ability of bacteria to form biofilm using the following formula: OD_570_ mean of the negative control plus three times the standard deviation (SD). The classification was as follows: non-biofilm formers (OD ≤ ODc), weak producers (ODc ≤ OD ≤ 2× ODc), moderate producers (2× ODc < OD), or strong producers (OD ≥ 4× ODc).

### 4.6. Antibiofilm Activity

The efficacy of PLA in inhibiting biofilm formation and eradicating pre-formed biofilms was evaluated using a previously described methodology [38].

The inhibition of biofilm formation was evaluated by adding increasing concentrations of PLA (ranging from 0.2 to 3 mg mL^−1^) to 96-well microplate wells containing 10^6^ CFU mL^−1^ of each bacterium, and after incubating plates at 37 °C for 24 h. After incubation, the staining method with crystal violet was performed, followed by microplate reading at the wavelength of 600 nm. The results were indicated as the percentage of biofilm formation inhibition as follows:

(1) $$Biofilm\;formation\;inhibition\;\%=\left(\frac{ODcontrol-ODassay}{ODcontrol}\;\right)\times 100\;$$

where ODcontrol corresponds to the mean optical density measured for bacterial biofilms grown in the absence of PLA, while ODassay is the mean optical density measured for bacterial biofilms grown in the presence of PLA.

The minimum biofilm inhibition concentration (MBIC) was defined as the lowest concentration of PLA capable of bacterial biofilm inhibition.

The influence of PLA on mature biofilms was assessed as follows: 24 h biofilms were treated with different concentrations of PLA (range, 0.2–3 mg mL^−1^), and the plates were further incubated at 37 °C for another 24 h. The supernatant was removed, and the total cell biomass was quantified by CV staining. The percentage of biofilm reduction was calculated using the following equation:

(2) $$Biofilm\;reduction\;\%=\frac{(AbsControl-AbsPLA)\;}{AbsControl}\times 100\;$$

The minimum eradication concentration (MBEC) was defined as the lowest concentration of PLA capable of eradicating mature bacterial biofilm.

### 4.7. Cytotoxicity Assays

The cytotoxicity of PLA was assessed in Caco-2 cells (Human Caucasian colon adenocarcinoma, ATCC collection) using the MTT assay. The cells were grown in Dulbecco’s modified Eagle’s medium (DMEM; Sigma-Aldrich, Saint Louis, MO, USA), supplemented with 1% penicillin–streptomycin, 1% L-glutamine, and 10% fetal bovine serum (Sigma-Aldrich, Saint Louis, MO, USA) at 37 °C with 5% CO_2_ in a humid environment. Cells were seeded at a density of 2 × 10^5^ cells per well in 96-well plates and incubated for 24 h. A concentration ranging between 0.25 and 2 mg mL^−1^ of PLA was used for cell treatment. Untreated cells were used as a negative control. After 24 h of treatment, 3-[4,5-dimethylthiazol-2-yl]-3,5 diphenyl tetrazolium bromide (MTT) solution was added to each well for 4 h at 37 °C. The resulting formazan crystals were solubilized with DMSO, and the absorbance was measured at 570 nm using a microplate reader.

### 4.8. Adhesion of Enteric Bacteria to Caco-2 Cells with PLA Treatments

To evaluate the ability of PLA to reduce the adhesion to cells, Caco-2 cells were seeded (2 × 10^5^ cells mL^−1^) onto 24-well plates and incubated for 24 h at 37 °C in 5% CO_2_ overnight. The cells were then treated with three different strains (10^8^ CFU mL^−1^) at a multiplicity of infection (MOI) of 100. The assays of adhesion on human cells were performed under pre-inoculation and post-inoculation conditions as reported previously [39].

In the pre-inoculation condition, the cells were treated with 1 mg mL^−1^ of PLA for 1.5 h and subsequently infected with each strain for 2 h; in the displacement assay, the cells were first infected with S1, S4, and E2, respectively, for 2 h, then treated with PLA at the same concentration, and further incubated for an additional 1.5 h. For both experiments, at the end of incubation, the infected monolayers were rinsed three times with PBS, serially diluted, plated on TSA agar plates and incubated for 24 h at 37 °C. The results were expressed as the percentage inhibition of the adhesion, with respect to controls performed in the same condition without the inoculation of PLA.

### 4.9. Transepithelial Electrical Resistance (TEER)

Caco-2 cells, grown as previously described, were seeded in the apical compartment of a transwell system (Falcon™ 6.4 mm diameter, 0.4 m pore size polyethylene terephthalate permeable Transwell filters, Corning) at a density of 3 × 10^5^/filter, and the medium was changed every 2 days. The cells were cultured for 21 days to ensure complete differentiation. Before the experimental phase, the monolayer integrity was evaluated by measuring the transepithelial electrical resistance (TEER) with a Millicell-ERS 3 voltohmmeter (Merck Millipore, Darmstadt, Germany) considering only those with the TEER higher than 300 Ohm cm^2^. Three different concentrations of PLA were tested (0.25, 0.5, and 1.5 mg mL^−1^) each in triplicate, and the measurement of the TEER was performed every hour for the first 3 h, and at the following 5–7–24 h from the treatment.

## 5. Conclusions

In conclusion, together with these results, PLA produced by lactic acid bacteria has shown strong antibacterial and antibiofilm activity against such foodborne pathogens as *Salmonella enterica* serotype Derby and *Escherichia coli* O26, which are known to form biofilms that enhance their persistence and resistance to treatments. It not only inhibits bacterial growth at low concentrations, but also disrupts biofilm formation, helping to reduce contamination and prevent infection spread. PLA’s non-toxic nature, especially toward human cells, makes it a promising option for food safety. Its ability to target both planktonic and biofilm-associated bacteria positions it as a valuable tool in managing foodborne illnesses. Overall, PLA offers a sustainable, natural solution for foodborne pathogen control, though further research is needed to fully understand its mechanisms and optimize its use, and future research should explore the long-term stability of PLA in different environmental conditions, as well as its efficacy in complex food matrices.

## Figures and Tables

**Table 1:** Antimicrobial susceptibility profiles of *S. enterica* Derby (S1), *S. enterica* Typhimurium DSMZ 18,522 (S4), and *E*. *coli* O26 (E2) against four antibiotics ampicillin (AMP), kanamycin (KANA), chloramphenicol (CHL), and tetracycline (TET). MIC values are interpreted based on EUCAST resistance cut-offs.

Strain	Antibiotic	Cut-Off R (EUCAST)	MIC	R/S
		(µg mL^−1^)		
S1	AMP	R > 8	>512	R
	KANA	R > 16	4	S
	CHL	R > 8	-	S
	TET	R > 8	4	S
S4	AMP	R > 8	128	R
	KANA	R > 16	4	S
	CHL	R > 8	4	S
	TET	R > 8	4	S
E2	AMP	R > 8	16	R
	KANA	R > 16	S ≥ 512	R
	CHL	R > 8	S ≥ 512	R
	TET	R > 8	16	R

Note: R: resistant; S: susceptible.

**Table 2:** Minimum inhibitory concentrations and minimum bactericidal concentrations of PLA against *S. enterica* Derby (S1), *S. enterica* Typhimurium DSMZ 18,522 (S4), and *E*. *coli* O26 (E2).

	MIC (µg mL^−1^)	MBC (µg mL^−1^)	MBC/MIC Ratio	
S1	2.0 ± 0.5	5.0 ± 0.5	2.5	bactericide
S4	2.5 ± 0.2	5.0 ± 0.5	2.0	bactericide
E2	2.75 ± 0.35	5.5 ± 0.25	1.81	bactericide

**Table 3:** Biomass in *S. enterica* Derby (S1), *S. enterica* Typhimurium DSMZ 18,522 (S4), and *E*. *coli* O26 (E2) biofilms (n = 3, mean ± SD) quantified by crystal violet staining and expressed as OD570. ODc = OD570 control + (3× SD control) = 0.06. Negative (OD ≤ ODc), weak (ODc ≤ OD ≤ 2× ODc), moderate (2× ODc < OD ≤ 4× ODc), and strong biofilm production (4× ODc < OD), according to Stepanović’s definition [23].

	Means (OD_570_) ± SD	Classification
S1	0.243 ± 0.011	Moderately forming biofilm
S4	1.904 ± 0.136	Strongly forming biofilm
E2	1.561 ± 0.282	Strongly forming biofilm

## Data Availability

The data presented in this study are available herein.
